# Supplementary material for: Commensal colonization of Candida albicans in the mouse gastrointestinal tract is mediated via expression of candidalysin and adhesins
Source: Microbiol Spectr. 2025 Jul 30;13(9):e00567-25. doi: 10.1128/spectrum.00567-25 (PMC12403622; doi:10.1128/spectrum.00567-25)
Supplement: Supplemental material — Fig. S1 to S4; Table S1. [file spectrum.00567-25-s0001.pdf]

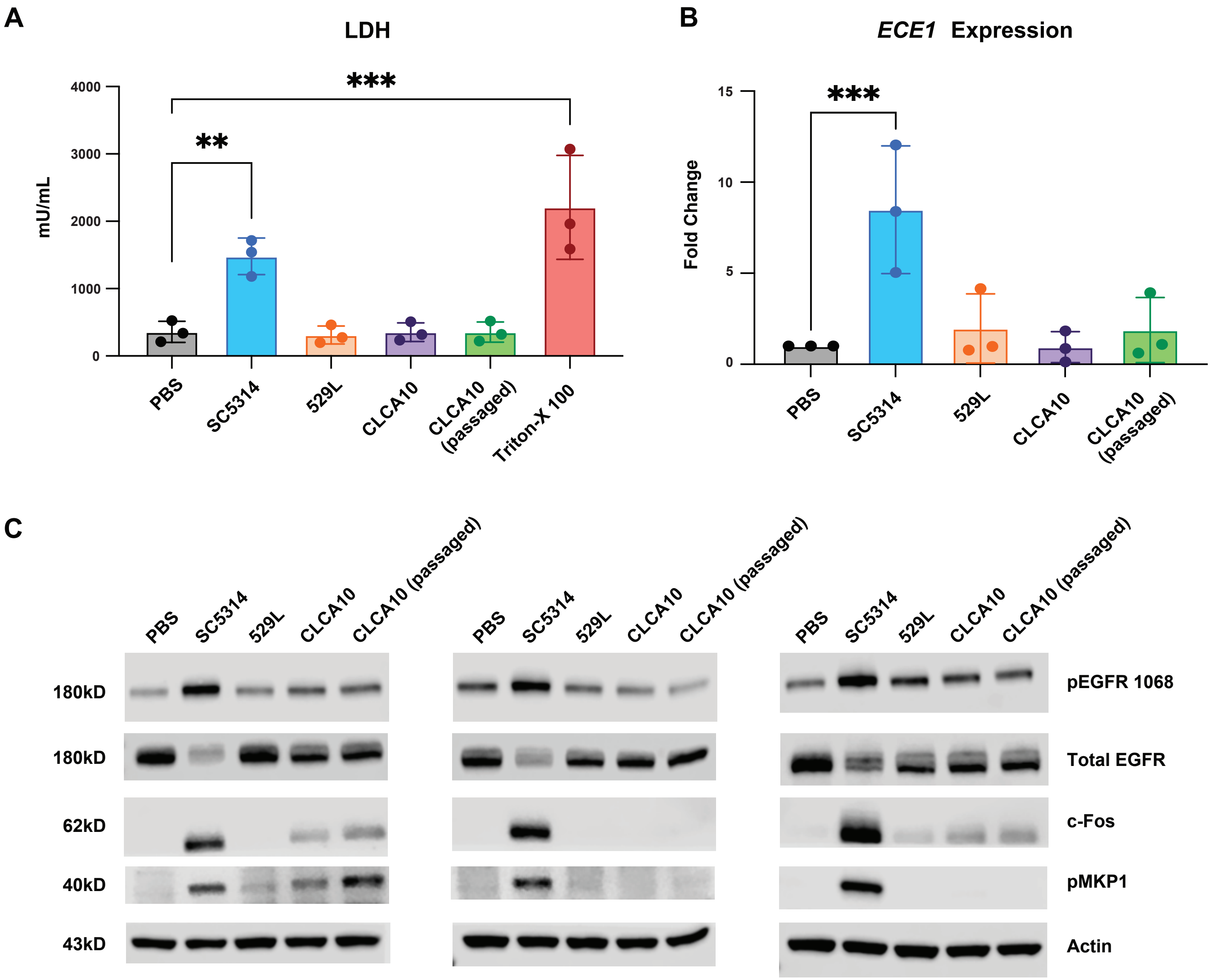

Supplemental Figure 1: CLCA10 and SC5314 exhibit different virulence phenotypes. A: mU/mL LDH mea-sured from TR146 cell supernatants after infection with *C. albicans* (MOI 0.1, 24 hours). B: Fold change in Ece1 RNA isolated from *C. albicans* after incubation with Tr146 cells (MOI 10, 4 hours). C: pEGFR 1068, total EGFR, c-Fos, pMKP1 and actin expression measured via western blotting from TR146 cell lysates after infection with *C. albicans* (MOI 10, 4 hours). Graphs display mean ± SD from 3 replicates per group. Significance determined using (A) one-way ANOVA with Dunnett's test and (B) repeated measures one-way ANOVA with Dunnett's test. ** = $p<0.01$, *** = $p<0.001$. Data representative of 2-3 experiments yielding comparable results.

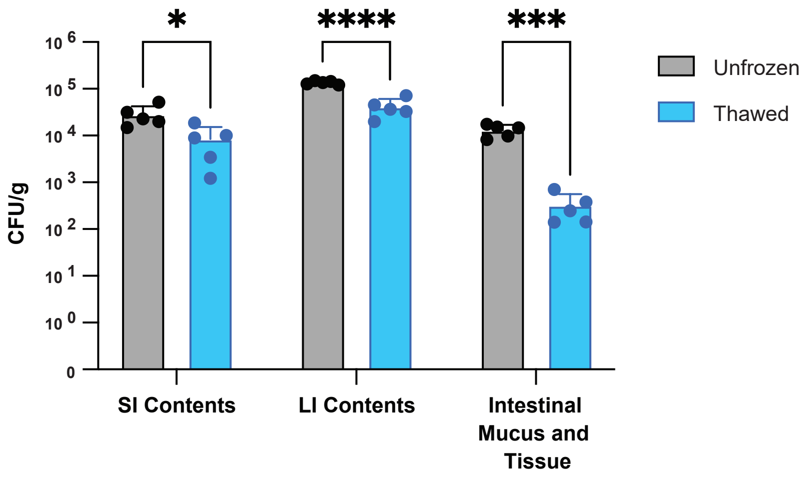

Supplemental Figure 2: Freezing samples results in reduced recovery via culturing. CFU/g *C. albicans* cultured from SI contents, LI contents, and intestinal tissue and mucus of mice infected with *C. albicans* (CLCA10) via oral gavage. Samples were either plated immediately after collection (Unfrozen) or frozen at -20°C and thawed before plating (Thawed). Graphs display mean ± SD from 5 mice per group. Significance determined using Student's t-test with Holm-Šídák correction. * = $p<0.05$, *** = $p<0.001$, **** = $p<0.0001$.

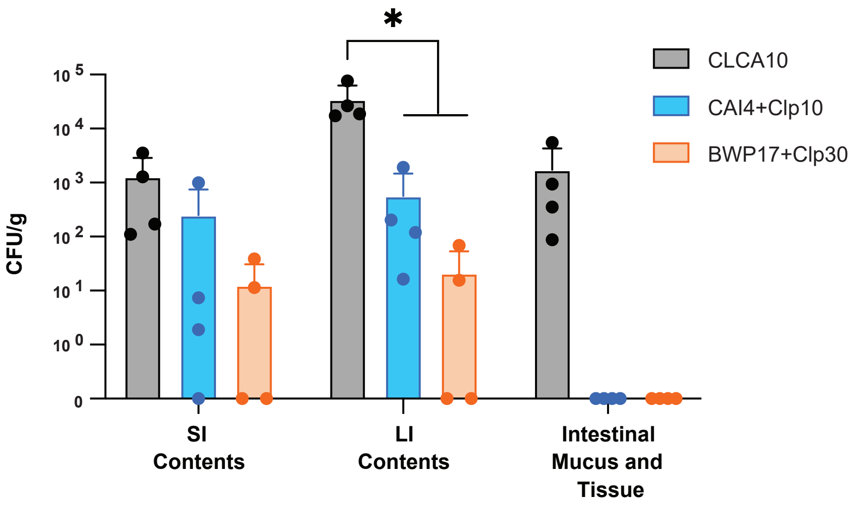

Supplemental Figure 3: Genetically modified SC5314-derived strains exhibit reduced colonization efficiency in the GI tract. CFU/g *C. albicans* cultured from SI contents, LI contents, and intestinal mucus and tissue of mice infected with C. albicans (CLCA10, BWP17+Clp30 or CAI4+Clp10) via oral gavage. Significance determined using one-way ANOVA with Dunnett's test for multiple comparisons. * = $p<0.05$. Data representative of 2 experiments yielding comparable results.

Gene	Sequence
	**pSNR52 R Sequence**
**ICL1**	GCTTCTGGGTTAGTGGCACCCAAATTAAAAATAGTTTACGCAAGTC
**CAT1**	TGATGGGTAGTTACCAGCAGCAAATTAAAAATAGTTTACGCAAGTC
**ECE1**	GTGGCAACACGAGCAACAATCAAATTAAAAATAGTTTACGCAAGTC
**HWP1**	GTTTCTACTGCTCCAGCCACCAAATTAAAAATAGTTTACGCAAGTC
**VPS51**	CGTTGAGACGGATTTCTTTCCAAATTAAAAATAGTTTACGCAAGTC
**ALS3**	AATCAGTGCCACCTGGTGGGCAAATTAAAAATAGTTTACGCAAGTC
**SAP2**	GTGGCAGCATCTGGAGAATTCAAATTAAAAATAGTTTACGCAAGTC
	**sgRNA Scaffold F Sequence**
**ICL1**	GGTGCCACTAACCCAGAAGCGTTTTAGAGCTAGAAATAGCAAGTTAAA
**CAT1**	CTGCTGGTAACTACCCATCAGTTTTAGAGCTAGAAATAGCAAGTTAAA
**ECE1**	ATTGTTGCTCGTGTTGCCACGTTTTAGAGCTAGAAATAGCAAGTTAAA
**HWP1**	GTGGCTGGAGCAGTAGAAACGTTTTAGAGCTAGAAATAGCAAGTTAAA
**VPS51**	GAAAGAAATCCGTCTCAACGGTTTTAGAGCTAGAAATAGCAAGTTAAA
**ALS3**	CCCACCAGGTGGCACTGATTGTTTTAGAGCTAGAAATAGCAAGTTAAA
**SAP2**	AATTCTCCAGATGCTGCCACGTTTTAGAGCTAGAAATAGCAAGTTAAA
	**SAT Deletion F**
**ICL1**	TACCTTTTATTCTAATATAAATTAAAGAATAAACATTAATAATATCTACCGGTACCGGGCCCCCCTCGAG
**CAT1**	TAGATCTTTTTTTTATTTTCAATTTCCTATTTATATATAAATTATTTATTGGTACCGGGCCCCCCTCGAG
**ECE1**	AACAAACAACTTTCCTTTATTTTACTACCAACTATTTTCCATTCGTTAAAGGTACCGGGCCCCCCTCGAG
**HWP1**	TCAAACACAACAGGAATCTCCTATAGTCACTCGCTTTTAGTTTCGTCAATGGTACCGGGCCCCCCTCGAG
**VPS51**	CAAGAAATCAAGAACTCAATTCACGTTTATTCCAAGCTCCCTTATATATAGGTACCGGGCCCCCCTCGAG
**ALS3**	AATTTCATTTTATTATAATTGTATAAACAACTACCAACTGCTAATATTAGGGTACCGGGCCCCCCTCGAG
**SAP2**	TCAATTAATCAATCAAATAACAACAACCCACTAAACATCACCATTTATCAGGTACCGGGCCCCCCTCGAG
	**SAT Deletion R**
**ICL1**	TATATTATAAGTTCTGTTTCTTTACTAATTTACACTTCTATCCCTCAAAACTCTAGAACTAGTGGATCT
**CAT1**	ACAATAGAAGAACTAATCTCAAATTAGCGCTTGCCTGGTTTCGTTCACACCTCTAGAACTAGTGGATCT
**ECE1**	TGGAATAAAAGATTAAGCTTGTGGAAAACAAATTTTTATCTGCTGAGCATCTCTAGAACTAGTGGATCT
**HWP1**	AAAAAAAGAAATCCCAAAAAAAGAACAAAACTTAGTATCAGTTATTAGAACTCTAGAACTAGTGGATCT
**VPS51**	AGTTGATGATATTCTATAAATATATCTATAAATGCGTAGGTAGATATGAACTCTAGAACTAGTGGATCT
**ALS3**	AAACAAACAAATAACAAAAATCTAAAAAGGCGACTATGATGGTATCATCCCTCTAGAACTAGTGGATCT
**SAP2**	AATCAAGCAACTAATATTTTAATATTTTAACTTTATTCCACCCCTTCATCCTCTAGAACTAGTGGATCT

Supplemental Table 1. Guide RNAs and repair constructs used to inactivate indicated genes (left column) in *Candida albicans* strain CLCA10. Underlined sequences indicate PCR primers for sequence amplification.

Source Data - Supplemental Figure 1C

pEGFR 1068

Replicates 1-2

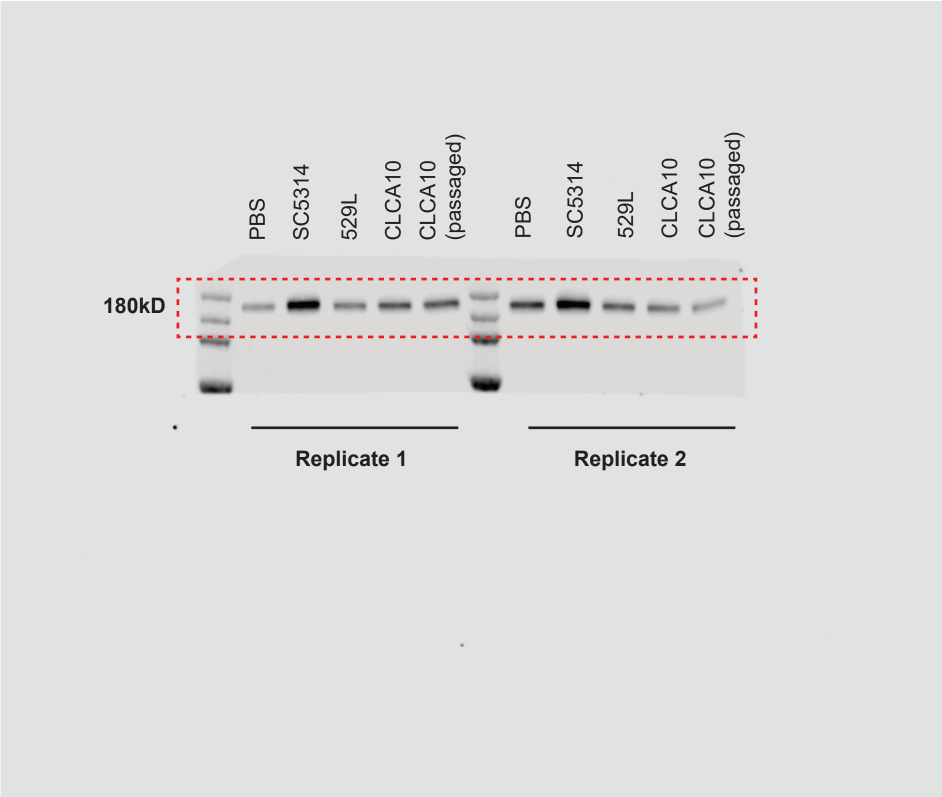

Replicate 3

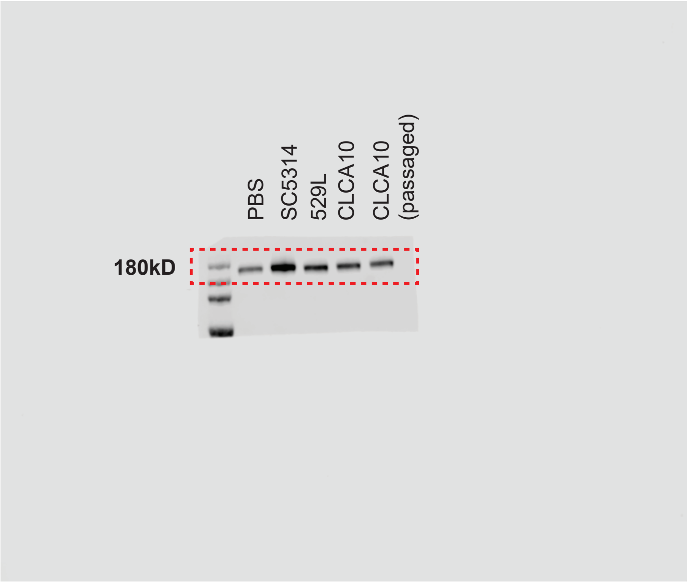

Source Data - Supplemental Figure 1C

Total EGFR

Replicates 1-2

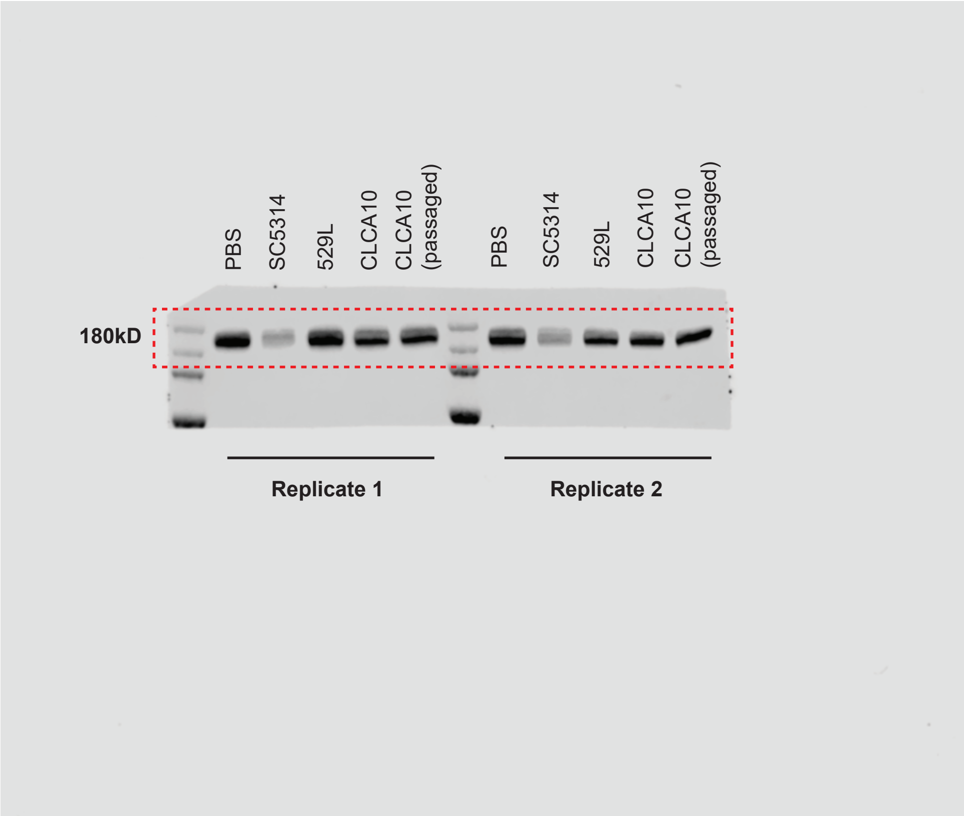

Replicate 3

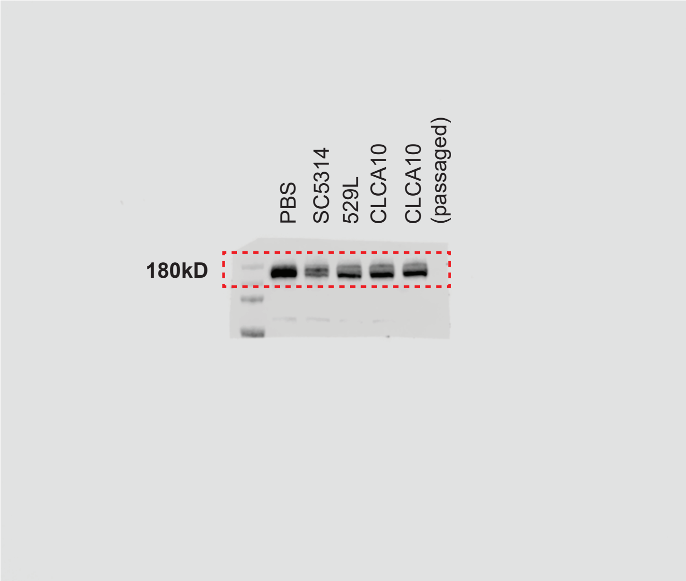

Source Data - Supplemental Figure 1C

c-Fos

Replicates 1-2

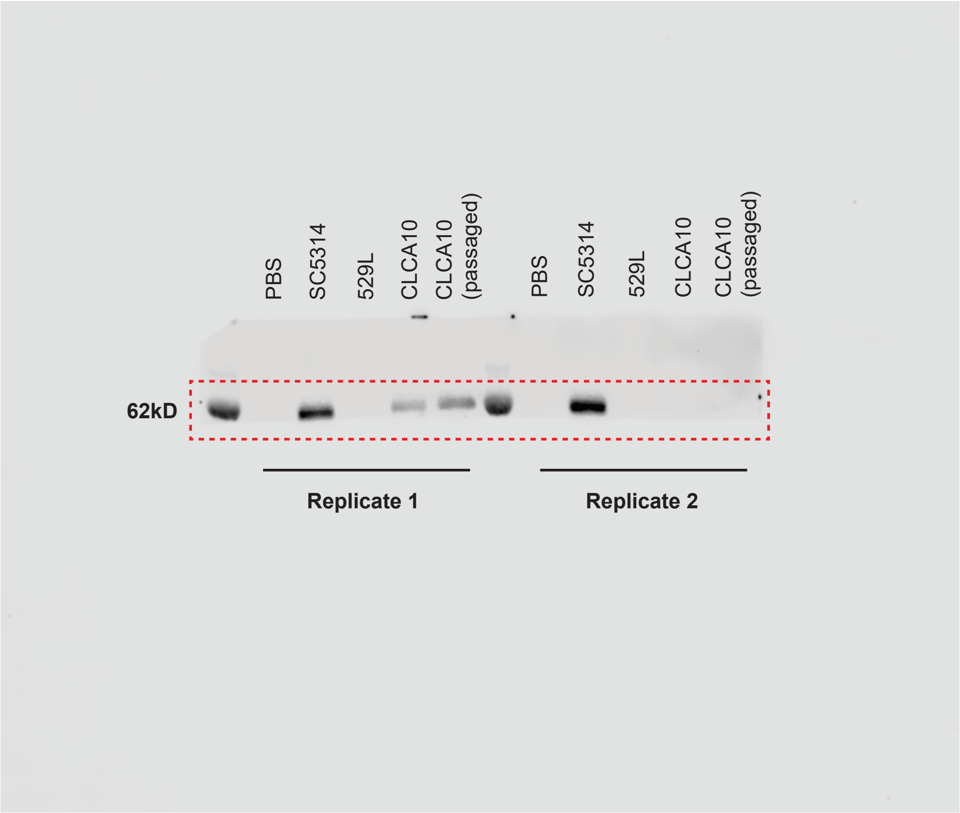

Replicate 3

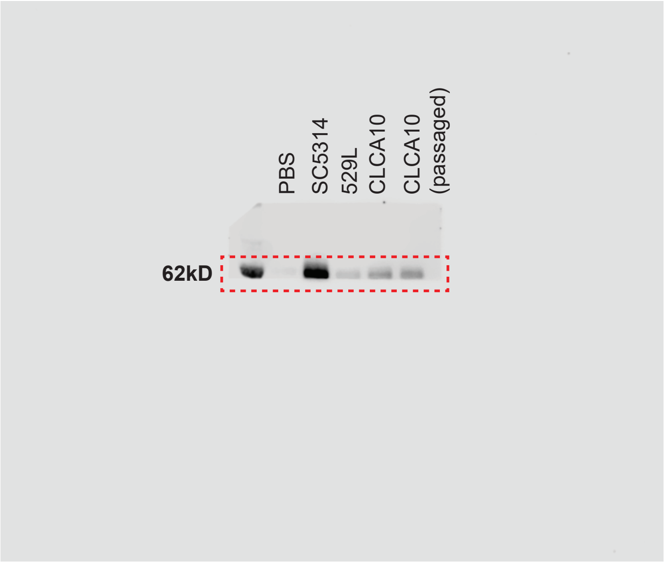

Source Data - Supplemental Figure 1C

pMKP1

Replicates 1-2

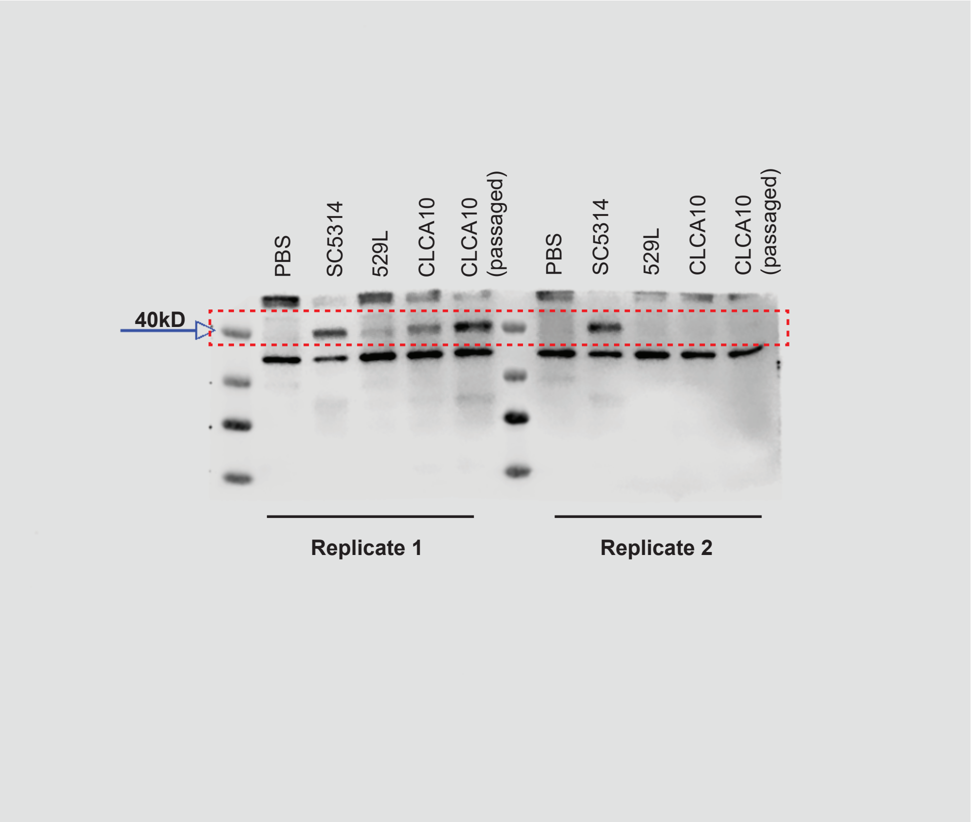

Replicate 3

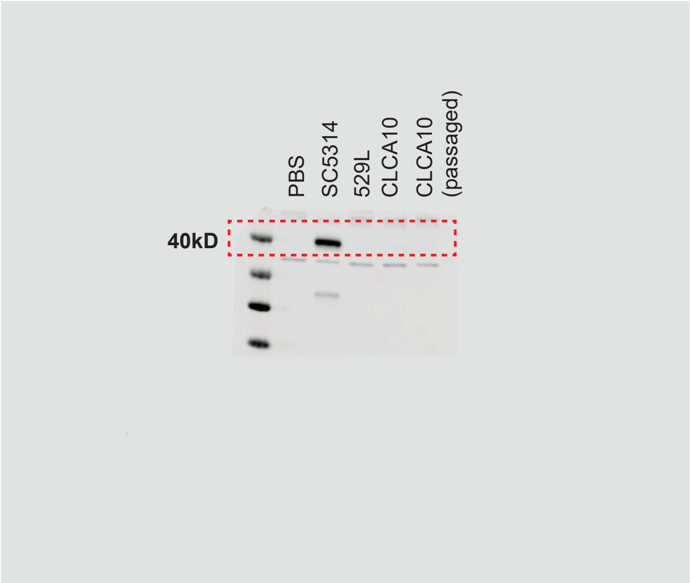

Source Data - Supplemental Figure 1C

Actin

Replicate 1

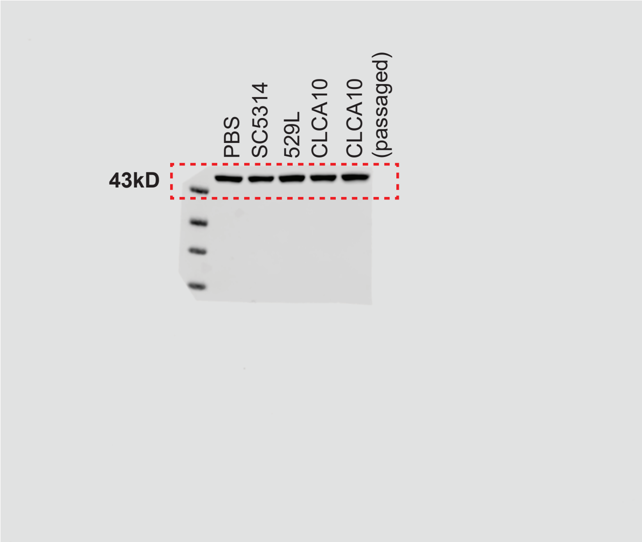

Replicate 2

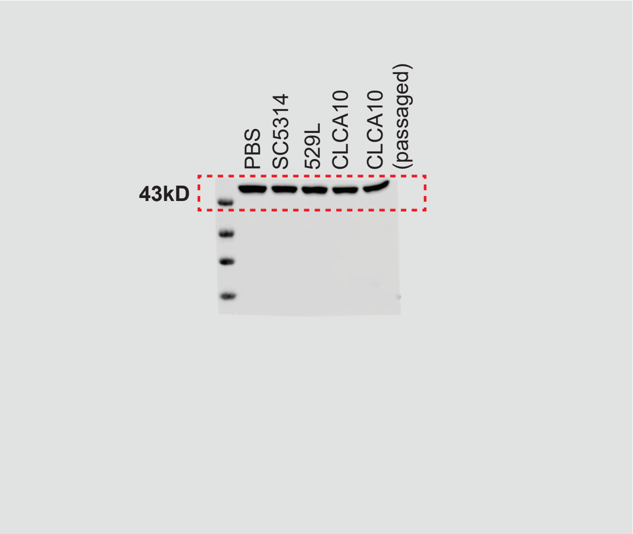

Replicate 3

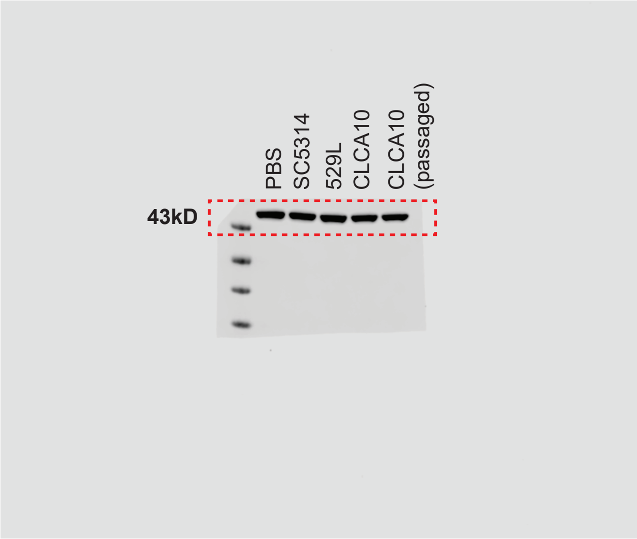

Supplemental Figure 4. Source blot images for Supplemental Figure 1C showing the three blots each for pEGFR 1068, Total EGFR, c-Fos, pMKP1, and Actin.
